# Multifunctional materials with potential antiviral applications in face masks, face shields, and hydrogels against mpox virus

**DOI:** 10.1038/s41598-025-17955-6

**Published:** 2025-09-01

**Authors:** Miguel Martí, Alba Cano-Vicent, Mercedes Cervera-Alamar, Rina Hashimoto, Kazuo Takayama, Ángel Serrano-Aroca

**Affiliations:** 1 Biomaterials and Bioengineering Lab, Department of Biotechnology, Universidad Católica de Valencia San Vicente Mártir, Valencia, 46001 Spain; 2 San Alberto Magno Translational Research Center, Universidad Católica de Valencia San Vicente Mártir, Valencia, 46001 Spain; 3 Department of Synthetic Human Body System, Medical Research Institute, Institute of Integrated Research, Institute of Science Tokyo, Tokyo, 1138510 Japan; 4 Center for iPS Cell Research and Application (CiRA), Kyoto University, Kyoto, 6068507 Japan

**Keywords:** Antimicrobial materials, Vaccinia virus, Mpox virus, Electrospun films, Multifunctional materials, Polymers, Hydrogels, Face masks, Face shields, Microbiology, Materials science

## Abstract

The recent emergence and global spread of the mpox virus (MPXV), formerly known as the monkeypox virus, underscores the urgent need for effective antiviral materials to combat this emerging zoonotic pathogen. This study evaluates the antiviral activity of five functional material films against vaccinia virus, a representative model of MPXV, by the TCID50 assay. The tested materials include two electrospun polyester fabrics functionalised with benzalkonium chloride (BAK) or soap, specifically designed for antiviral face masks. Three other material films were also tested: two biocompatible hydrogels composed of alginate crosslinked with Ca^2+^ and Zn^2+^ or acetic acid-loaded chitosan, and a polyethylene terephthalate (PET) film coated with BAK, developed for used in face shields. All materials showed significant antiviral activity (≥ 87.85% viral inactivation): the polyester-BAK and polyester-soap films achieved 90.00% and 87.85%, respectively; the alginate-based and chitosan-based films reached 92.49% and 89.20%, respectively; and the PET-BAK film showed the highest efficacy (94.45%). These findings report on the potential antiviral activity of these materials against MPXV and highlight their applications in protective equipment and hydrogel-based medical treatments to combat this pathogen and other emerging microbial threats, including those related to bioterrorism or microbial warfare.

## Introduction

A member of the *Poxviridae* family of the genus *Orthopoxvirus*, the mpox virus (MPXV), previously referred to as the monkeypox virus, is divided into two separate clades: clade I, which includes subclades Ia and Ib, and clade II, which comprises subclades IIa and IIb^1^. Since 2022, the World Health Organization (WHO) has declared two Public Health Emergencies of International Concern (PHEICs) related to mpox. The first was announced in July 2022 and ended in May 2023^2^. Due to the emergence of a new mpox strain and its growing transmission in Central and East Africa, a second PHEIC was initiated in August 2024 and remains in effect^1^. While one virus strain (clade IIb) continues to circulate within the WHO European Region, new virus strains (clades Ia and Ib) have primarily been reported as imported cases.

This zoonotic virus, closely related to the variola virus that causes smallpox, spreads through direct contact with infected individuals, respiratory droplets, and contaminated surfaces^3,4^underscoring the urgent need for developing effective antiviral materials to reduce its transmission risks in healthcare and public environments^5,6^. Recent studies suggest that MPXV can persist on surfaces for extended periods, further increasing the risk of indirect transmission, particularly in healthcare settings and high-contact environments^7^.

In response to the COVID-19 pandemic, a wide range of antimicrobial materials have been developed^8–15^. Several of these materials have shown effective antiviral activity against enveloped viruses such as SARS-CoV-2^6,16–24^: electrospun polyester fabrics functionalised with benzalkonium chloride (BAK)^20^ or solidified hand soap^21^designed for use in low-cost antiviral face mask with enhanced protection; two hydrogel films, one composed of alginate crosslinked with Ca²⁺ and Zn²⁺^22^ and another made of chitosan/acetic acid^23^; and a BAK-coated polyethylene terephthalate (PET) film designed for antiviral face shields^24^.

Given that MPXV is also an enveloped virus, we hypothesised that these five antiviral material films, previously proven effective against other enveloped viruses, could exhibit significant antiviral potential against this pathogen. In this study, vaccinia virus (VACV) was used as a representative model of MPXV due to their close genetic relationship as double-stranded DNA viruses of the *Orthopoxvirus* genus^25,26^both classified within Baltimore group I^27^. Furthermore, VACV can be handled under Biosafety Level 2 conditions, in contrast to MPXV, which requires Level 3 containment.

To the best of our knowledge, this is the first report describing five multifunctional materials with potential antiviral activity against mpox virus, which can be integrated into face masks, face shields, and therapeutic hydrogels.

## Results

**Fig. 1:**
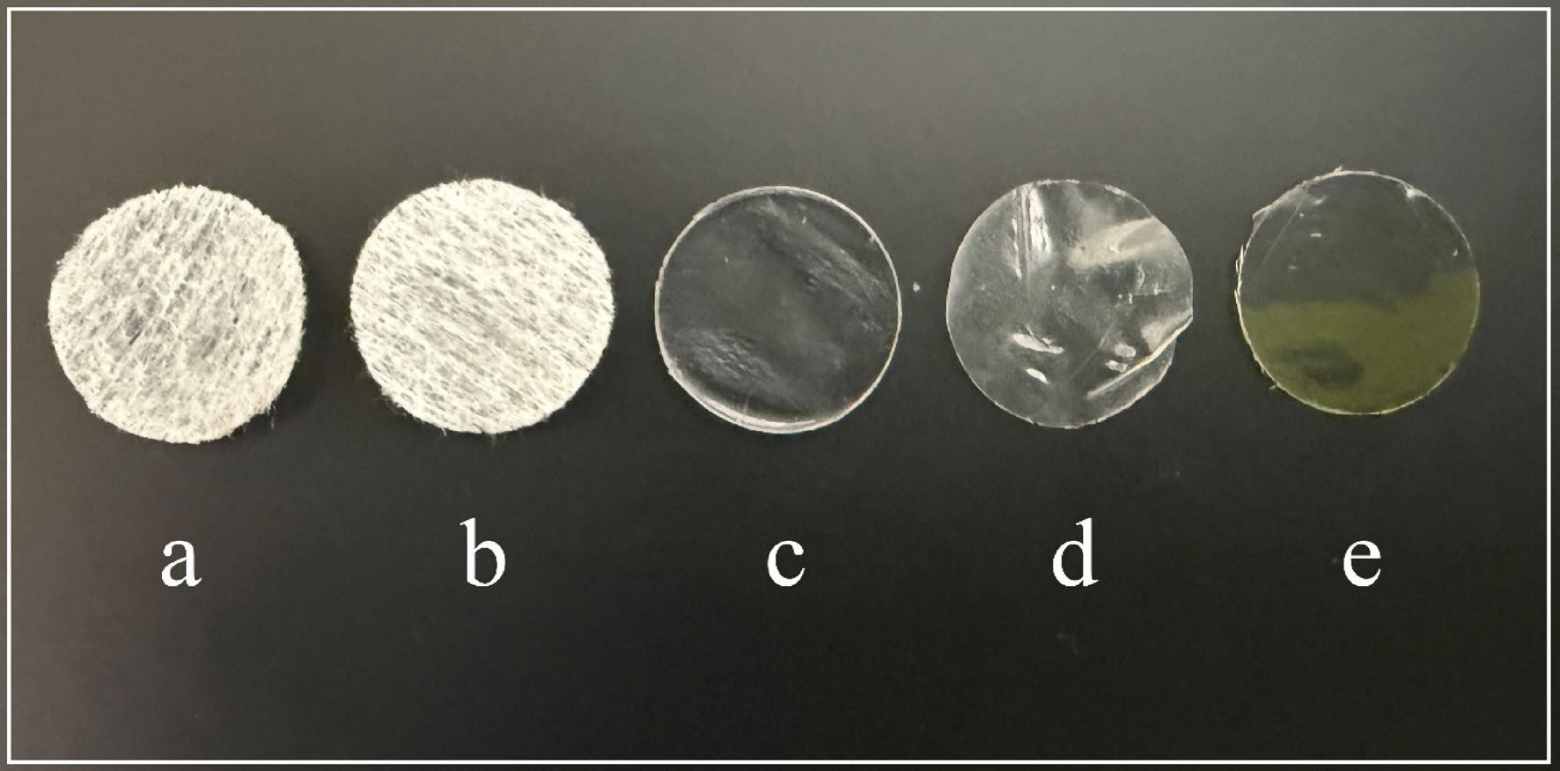
Tested material films against vaccinia virus: (**a**) Electrospun polyester/benzalkonium chloride; (**b**) Electrospun polyester/solidified hand soap; (**c**) Polyethylene terephthalate/benzalkonium chloride; (**d**) Alginate crosslinked with Ca²⁺ and Zn²⁺; (**e**) Chitosan/acetic acid. *Photographs of the materials taken by the authors.*

The five multifunctional materials studied here were previously synthesized and characterized by our research group^20–24^. Figure 1 shows the photographs of the five material films tested against VACV in this study.

The antiviral properties of these five material films were tested against the vaccinia virus, a widely accepted surrogate for the mpox virus, to evaluate their potential use in advanced biomedical applications such as antiviral face masks, face shields and therapeutic hydrogels aimed at combating this emerging pathogen. Table 1 presents the antiviral results of the five functional materials (Fig. 1a and e) against VACV, expressed as the median tissue culture infectious dose per mL (TCID50/mL), percentage of viral inactivation and log reduction of TCID50/mL relative to the control (virus that has not been exposed to any material).

**Table 1:** Infectious titers of vaccinia virus after 24 h of viral contact with five advanced materials measured by the TCID50/mL assay. Antiviral results are shown as TCID50/mL values (mean ± standard deviation), % viral inactivation and log reduction of TCID50/mL with respect to the control (virus that has not been exposed to any material). Statistical differences compared to the control sample are indicated: ****p < 0.0001.

Control	TCID50/mL		
Virus that had not been exposed to any material	3.82∙10^6^±5.99∙10^5^		
Multifunctional material film	TCID50/mL	% Viral inactivation	Log reduction of TCID50/mL
Electrospun polyester/benzalkonium chloride	3.82∙10^5^±5.99∙10^4^****	90.00	1
Electrospun polyester/solidified hand soap	4.64∙10^5^±8.65∙10^4^****	87.85	0.92
Polyethylene terephthalate/ benzalkonium chloride	2.12∙10^5^±9.16∙10^4^****	94.45	1.28
Alginate crosslinked with Ca^2+^ and Zn^2+^	2.87∙10^5^±1.31∙10^5^****	92.49	1.15
Chitosan/acetic acid	4.13∙10^5^±8.83∙10^4^****	89.20	0.97

These results show that the electrospun polyester fabrics functionalized with BAK and soap exhibit significant antiviral activity (90 and 87.85% viral inactivation, respectively) against VACV, in good agreement with its previously demonstrated efficacy against other enveloped viruses^20,21^.The PET/BAK film showed the highest antiviral activity against VACV, achieving 94.45% viral inactivation. Both hydrogel films, one composed of alginate crosslinked with Ca²⁺ and Zn²⁺^22^and the other based on chitosan/acetic acid^23^also showed significant antiviral effects against VACV, with viral inactivation rates close to or exceeding 90%.

## Discussion

**Fig. 2:**
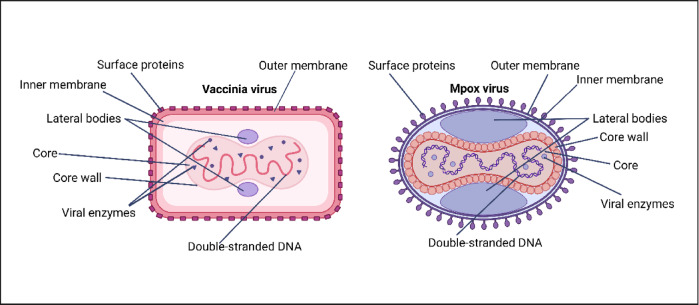
Structural comparison of vaccinia virus (VACV) and mpox virus (MPXV). *Figure created by Á.S-A and M.M using BioRender.*

Five advanced materials, previously shown to be effective against enveloped viruses such as SARS-CoV-2, were evaluated against another enveloped virus, VACV, a widely used representative model of MPXV (Fig. 2).

While VACV is primarily used as a vaccine vector and MPXV is a zoonotic virus responsible for mpox disease, both share similar structural features, including a brick-shaped morphology, a dumbbell-shaped core containing a double-stranded DNA genome, and two lateral bodies^25,26^.

The antiviral mechanisms of materials targeting enveloped viruses are usually attributed to disrupting the integrity of the viral lipid membrane, inactivating viral proteins, or generating unfavourable environmental conditions that hinder viral survival and replication. Several studies have demonstrated the efficacy of various functionalised materials against enveloped viruses like SARS-CoV-2, which share structural similarities with other pathogens such as MPXV and VACV. For instance, electrospun polyester/BAK filters and electrospun polyester/solidified hand soap films (Fig. 1a and b) effectively degrade the viral envelope and disrupt viral integrity or may destabilise the lipid bilayer of enveloped viruses, leading to their inactivation^20,21^. Lastly, PET films have also demonstrated strong antiviral properties after incorporating an antimicrobial BAK coating (Fig. 1c)^24^. Biopolymer-based films, such as alginate hydrogels crosslinked with Ca²⁺ and Zn^2+^ (Fig. 1d), offer antiviral activity through metal ion interactions that impair viral surface proteins and hinder infectivity^22^. Lastly, chitosan hydrogel films containing acetic acid (Fig. 1e) may further contribute to viral inactivation by modifying the local pH, creating an unfavourable environment for viral stability^23^. This material gradually releases acetic acid into the surrounding environment, potentially inducing localized acidification that contributes to viral inactivation by compromising envelope integrity and promoting the denaturation or dissociation of surface glycoproteins critical for viral attachment and entry, thereby reducing infectivity^28,29^.

**Fig. 3:**
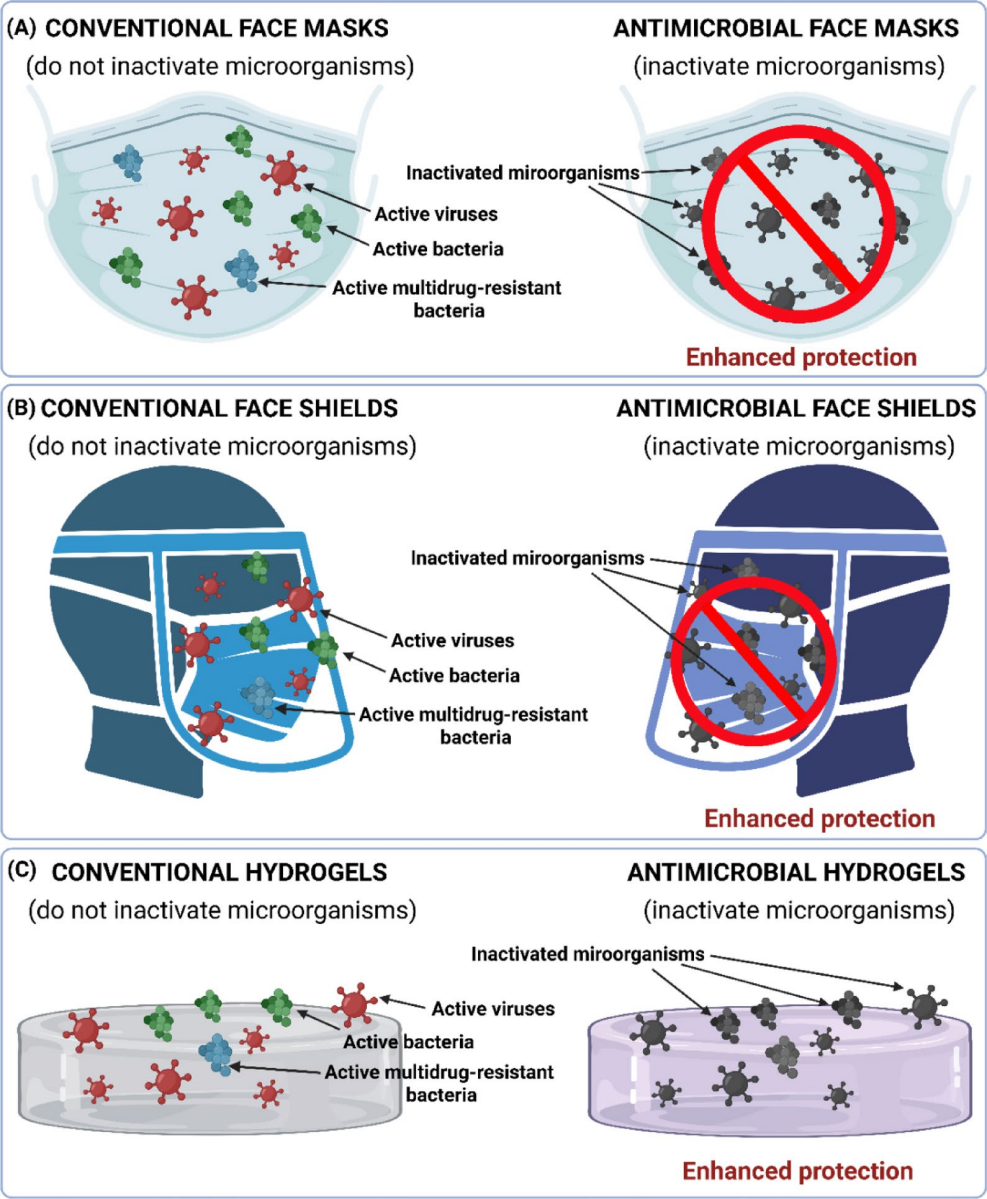
Antimicrobial face masks, antimicrobial face shields and antimicrobial hydrogels provide enhanced protection against microbial infections: (**A**) Conventional face masks versus antimicrobial face masks; (**B**) Conventional face shields versus antimicrobial face shields; (**C**) Conventional hydrogels versus antimicrobial hydrogels. *Figure created by Á.S-A and M.M using BioRender*.

The antimicrobial electrospun polyester/BAK filter was specifically developed by our research group for incorporation into face masks during the COVID-19 pandemic and was later exploited at industrial level by Visor Medical S.L., a division within Visor Fall Arrest Nets S.L. from Alicante, Spain^16^ It was designed to provide enhanced protection against SARS-CoV-2, along with antibacterial activity against multidrug-resistant bacteria, including methicillin-resistant *Staphylococcus aureus* (MRSA) and *Staphylococcus epidermidis* (MRSE)^20^. This antimicrobial face mask addresses a key limitation of conventional face masks, which primarily serve as physical barriers to block microorganisms from entering the respiratory system but are not made of functional materials that effectively inactivate them (Fig. 3A).

Other effective antiviral materials against enveloped viruses, used in antiviral face mask fabrics, were produced by functionalising an electrospun polyester film with solidified hand soap^21^. This functional material also provided significant antiviral activity (87.85% viral inactivation) against VACV (Table 1), as it had previously provided against other enveloped viruses^21^. The BAK and hand soap-based technology presents the advantage of being low-cost. Their affordability and ease of production make them highly suitable for large-scale industrial implementation and accessible for use in resource-limited settings, including low- and middle-income countries. These technologies could be used to enhance protection by manufacturing face masks (Fig. 3A) and other infection-prevention clothing (e.g. caps, scrubs, shirts, trousers, disposable gowns, overalls, hoods, aprons, and shoe covers), in the event of a mpox pandemic or in regions where the virus is severely impacting public health. The PET/BAK film showed the highest antiviral activity (94.45% viral inactivation) against VACV. The BAK technology applied to PET films can be used to manufacture protective face shields^24^. This material also showed potent antiviral activity against other enveloped viruses such as SARS-CoV-2, and antibacterial activity against multidrug-resistant bacteria such as MRSA and MRSE. Unlike conventional face shields, which primarily act as physical barriers against droplets and airborne particles, this next-generation antimicrobial face shield incorporates active components capable of inactivating microorganisms, addressing a key limitation of standard designs (Fig. 3B).

On the other hand, the two hydrogel films, one composed of alginate crosslinked with Ca²⁺ and Zn²⁺^22^ and the other of chitosan/acetic acid^23^showed previously antiviral activity against both enveloped (bacteriophage phi 6) and non-enveloped (bacteriophage MS2) viruses in good agreement with the significant antiviral activity observed here against VACV (very close or higher than 90% viral inactivation). These results suggest that these hydrophilic materials may possess broad-spectrum antiviral properties and their antiviral mechanisms may extend beyond interactions with lipid envelopes. For instance, mechanisms involving metal ion release, oxidative stress, or electrostatic interactions may contribute to virus inactivation even in the absence of an envelope. Nevertheless, further studies are needed to fully understand these inactivation mechanisms and determine their efficacy across different virus types. Both biodegradable and sustainable hydrogels (Fig. 3C) showed in vitro biocompatibility in human keratinocyte HaCaT cells^22,23^while the alginate-based hydrogel also demonstrated in vivo biocompatibility and antibacterial activity against MRSE^22^.

These findings reinforce the potential of these material films for biomedical applications in environments in which viral infections such as mpox pose significant health risks.

The antiviral performance of these materials may also be influenced by their surface topography and physicochemical characteristics. The five material films evaluated in this study display distinct morphologies and physicochemical features, including differences in porosity, pore size, and viscoelasticity, which are likely to affect the mechanisms of viral inactivation^30^. However, the present work was primarily aimed at demonstrating the potential of these materials to inactivate MPXV, rather than elucidating their precise mechanism of action. Future studies should seek to establish correlations between structural and mechanical parameters and antiviral efficacy.

## Methods

### Material films synthesis

Disk-shaped film specimens (approximately 10 mm in diameter) were prepared from the five types of materials analysed in this study by dry cutting using a cylindrical punch. All the disks were dried at 60 °C for 48 h until reaching constant weight and subsequently sterilized under UV light for one hour on each side before antiviral testing.

The electrospun polyester fabric films were purchased from NV EVOLUTIA (Valencia, Spain)^20,21^. A set of these electrospun polyester fabric films (*n* = 3) were functionalized with BAK^20^ by the dip coating method^31^. The films were thus immersed in a commercial 70% ethyl alcohol solution containing 0.1% *w/w* benzalkonium chloride (Montplet, Barcelona, Spain) for 1 min at 25 °C. This process resulted in a final dry BAK content of 0.46 ± 0.13% *w/w*.

Another set of NV EVOLUTIA electrospun polyester fabric films (*n* = 3) were functionalized with solidified hand soap^21^ by the dip coating method. The films were thus immersed in a highly diluted (1% *w/v*) aqueous solution of a commercial liquid hand soap (KYREY dermo, Laboratorios Forenqui, S.A., Picassent, Valencia, Spain) for 30 min at 23 ± 1 °C, resulting in a low dry hand soap content of 0.57 ± 0.03% *w/w*.

PET films were also functionalised with BAK^24^ by the dip coating method. Thus, PET sheets, with a thickness of 0.3167 ± 0.0408 mm and typically used in the production of commercial face shields, were sourced from Plásticos Villamarchante S.L. (Valencia, Spain)^32^. From these transparent PET sheets, three disk-shaped specimens (*n* = 3), each approximately 10 mm in diameter, were prepared and treated by immersion in a 70% ethyl alcohol solution containing 0.1% *w/w* BAK (Montplet, Barcelona, Spain) for 1 min at 25 °C, resulting in a dry BAK loading of 0.182 ± 0.034% *w/w*.

Characterization of the BAK compound used in the electrospun polyester/BAK and PET/BAK films was previously performed by nuclear magnetic resonance (NMR) spectroscopy^20^. The corresponding 1D proton NMR spectrum is shown in Fig. 1 of that previous study.

Hydrogel films of alginate were produced by crosslinking with Ca^2+^ and Zn^2+^^22^. Sodium alginate, calcium chloride and zinc chloride were purchased form Sigma-Aldrich (Saint Louis, MO, USA). The sodium alginate used to prepare these films was previously characterized by ^1^H-NMR spectroscopy^18^ by the ASTM protocol^33^. The results of this analysis revealed the distribution and sequence arrangement of guluronic acid (G) and mannuronic acid (M) residues in the alginate polymer. The overall composition was found to be 46.3% guluronic acid and 53.7% mannuronic acid, corresponding to an M/G ratio of 1.16. The dyad frequencies, indicating the probability of specific pairwise sequences, were as follows: FGG = 0.282, FGM = FMG = 0.181, and FMM = 0.356. The occurrence of specific triad sequences was also determined: FGGM = FMGG = 0.048, and FGGG = 0.234. These values suggest that guluronic acid residues are predominantly found in relatively short consecutive G-blocks, with an average length of approximately 7 residues.

To prepare the crosslinked alginate films, the sodium alginate (0.25 g) was dissolved in 30 mL of distilled water under magnetic stirring for 1 h at 24 ± 0.5 °C. The resulting solution was poured into a Petri dish and left to rest at room temperature for 24 h, followed by incubation at 37 °C for 48 h to allow film formation. Separately, a crosslinking solution was prepared by dissolving calcium chloride (5 g) in 500 mL of distilled water with magnetic stirring for 15 min at 24 ± 0.5 °C. The alginate film was then immersed in this calcium chloride solution for 1 h at 24 ± 0.5 °C to induce crosslinking and subsequently rinsed three times with distilled water. To prepare the second crosslinking solution, zinc chloride (0.1 g) was dissolved in 500 mL of distilled water. The films were immersed in the zinc chloride solution for 2 h at 24 ± 0.5 °C, then rinsed three times with distilled water. After that, they were transferred to Petri dishes and dried at room temperature for 24 h to allow slow solvent evaporation without film cracking, followed by an additional 48 h at 37 °C to ensure complete dehydration.

The chitosan films were produced by solvent casting using acetic acid as solvent^23^. A 10% *v/v* acetic acid solution was prepared by diluting acetic acid in distilled water. Chitosan (0.25 g) was then dissolved in 30 mL of this solution using magnetic stirring for 1 h at 24 ± 0.5 °C. The resulting chitosan solution was poured into a Petri dish and left at room temperature for 24 h to allow slow solvent evaporation and prevent film cracking. The Petri dish was then transferred to an oven and maintained at 37 °C for 48 h to ensure complete drying. The acetic acid content of the films was quantified gravimetrically, yielding a result of 15.39 ± 1.76% weight of 10% *v/v* acetic acid solution present in the chitosan film.

The chitosan used for film preparation is derived from crab shells (high viscosity, Product No. 48165, Lot # BCBP6349V) and was obtained from Sigma-Aldrich (Saint Louis, MO, USA). This chitosan had been previously characterized and exhibited a molecular weight of 183 kDa^34^. Acetic acid (purity ≥ 99.8%) was supplied by Honeywell and purchased through Sigma-Aldrich (Saint Louis, MO, USA).

### Vaccinia virus preparation

Vaccinia virus strain LC16m8 provided by National Institute of Infectious Diseases was used in this study under biosafety level 2 (BSL-2) conditions at Kyoto University. VACV were replicated in rabbit kidney epithelial (RK13) cells (Cat# RCB0183, RIKEN Cell Bank). After confirming cytopathic effects (CPE) in RK-13 cells infected with VACV, the culture supernatant and floating cells were collected and centrifuged at 2,000 rpm for 5 min. The supernatant was then discarded, and 2.5 mL of fresh medium was added to the resulting cell pellet. The cells were disrupted by performing two freeze-thaw cycles. The resulting cell suspension was passed through a 40 μm strainer to remove debris, and the clarified viral solution was stored at -80 °C. RK13 cells were cultured with Dulbecco’s Modified Eagle Medium (DMEM) (Cat# 043-30085, FUJIFILM Wako Pure Chemical) supplemented with 10% fetal bovine serum and 1% penicillin/streptomycin.

### Viral titration

The antiviral activity of the multifunctional material films was evaluated after 24 h of viral contact. Viral titers were measured by the median tissue culture infectious dose (TCID50) assay^6,18,24^. A volume of 100 µL of 10⁷ TCID50/mL VACV was applied to the material films and incubated at 37 °C for 24 h. Subsequently, 900 µL of medium was added to recover the virus, which was then subjected to the TCID50 analysis.

RK13 cells (Cat# RCB0183, RIKEN Cell Bank) were seeded into 96-well cell culture plates (Cat# 167008, Thermo Fisher Scientific). Samples were serially diluted 10-fold from 10^− 1^ to 10^− 8^ in cell culture medium, transferred onto the cells, and incubated at 37 °C for 96 h. Cytopathic effects were evaluated under a microscope. TCID50/mL was calculated using the Reed-Muench method^35^. These experiments were performed using three replicates for each type of material.

The results are expressed as TCID50/mL (mean ± standard deviation), percentage of viral inactivation (% viral inactivation, *I%*), and log reduction of TCID50/mL with respect to the control. The control consisted of virus samples that had not been exposed to any material. The percentage of viral inactivation (*I%*) was calculated using the following equation, where the mean TCID50/mL of the tested *sample* (*n* = 3) was compared to that of the *control* (*n* = 3):

$$\:I\%=100-\left(\frac{\:Sample\:\bullet\:100}{Control\:}\right)$$

Log reduction was determined by subtracting the mean log (TCID50/mL) of the tested sample (*n* = 3) from that of the control (*n* = 3).

### Statistical analysis

Antiviral assay data were analysed using one-way ANOVA followed by Tukey’s post hoc test. Statistically significant differences were identified at ****p < 0.0001, using GraphPad Prism 10.

## Conclusions

This study identified five advanced materials with significant antiviral activity against the vaccinia virus: two electrospun polyester films functionalised with BAK or solidified hand soap, two biocompatible hydrogels composed of alginate crosslinked with Ca^2+^ and Zn^2+^ or chitosan/acetic acid, and a PET film coated with BAK. Therefore, these materials could be potentially useful in combating a potential mpox pandemic, or in regions where the virus is severely affecting public health, and provide defence against other emerging microbial threats, including those arising from a microbiological warfare. They could be included in the production of protective equipment such as face masks, face shields and antiviral hydrogels for medical treatments and prevention of mpox virus. However, further studies are needed to explore the scalability of these functional materials for widespread use.

## Data Availability

Raw data of the antiviral results can be obtained from the corresponding authors or can be accessed at the following repository [https://doi.org/10.6084/m9.figshare.28731062].
